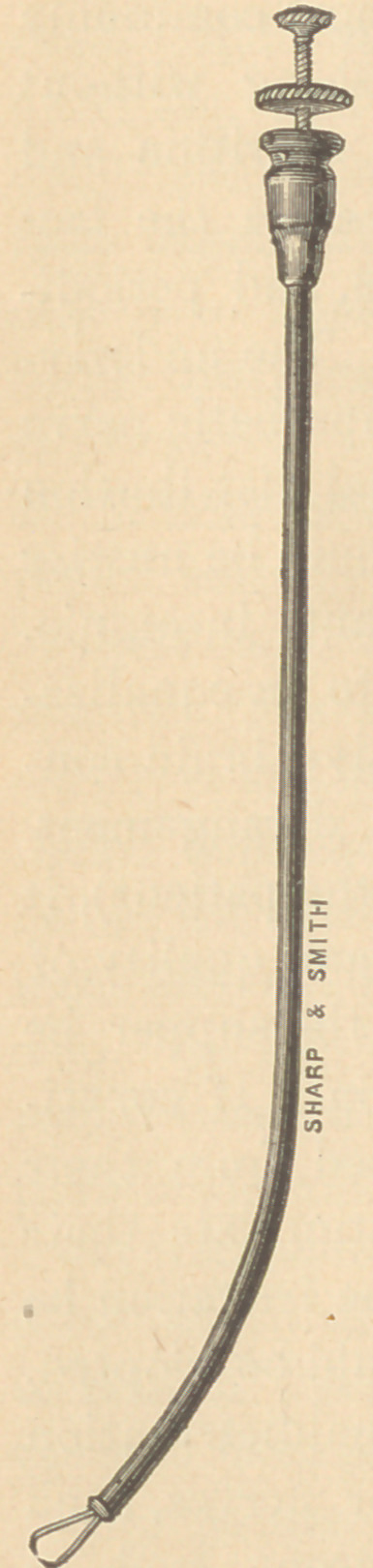# Laryngeal Sponge Holder and Forceps

**Published:** 1877-01

**Authors:** Frank H. Davis

**Affiliations:** Chicago


### A LARYNGEAL SPONGE HOLDER AND FORCEPS.

By FRANK H. DAVIS, M.D., Chicago.

The Laryngeal sponge and brush holder shown in the accompanying cut is formed of a hard rubber tube, the size and length of a catheter, conveying through its center a small steel staff. This staff is split at the end with a slightly hooked fork, which spreads as the staff is thrust out from the tube, and is closed or clamped together as it is withdrawn in again. A bit of sponge or the brush from a camel's hair pencil, placed in the fork and the staff withdrawn, is hooked through and tightly drawn against the end of the instrument. The nut at the handle can then be turned up against the end of the tube, and thus prevent any possibility of the brush or sponge becoming detached or dropping into the trachea. The sponge is also readily discharged without the necessity of soiling the fingers, and a fresh one substituted for each application. The instrument has a perfectly smooth even end, with no bulbous enlargement to be caught in the glottis; the rubber hook is also very much better tolerated in its contact with the mucous membrane of the throat than the ordinary metal holders. By slight heat this curve of the instrument can be adjusted to any angle desired. It is thus rendered available in making applications to the pharynx, anterior or posterior nares, and also for plugging the posterior nares. In this latter operation a silk thread is tied to a bit of sponge caught in the holder, and is passed through the side of the nose to be plugged. The staff being pressed outward will appear with the sponge below the soft palate, and can be seized by a forceps, the sponge detached, and the holder withdrawn. The plug is then attached to the cord, as ordinarily directed in this operation. The instrument is also available as a light laryngeal or nasal forceps for removing small foreign bodies. Its use for this latter purpose must be directed by the laryngeal mirror, and it is consequently not as readily used as the hair snare or probang.

## Figures and Tables

**Figure f1:**